# An Artificial Anion Channel Based on Supramolecular Calix[4]Pyrrole and Its Application as an Anti‐Cancer Agent

**DOI:** 10.1002/asia.70905

**Published:** 2026-07-21

**Authors:** Hidekazu Miyaji, Yuki Shiozawa, Takahiro Matsubara, Tomoyoshi Terada, Yoshinori Muto

**Affiliations:** ^1^ Department of Chemistry and Biomolecular Science Faculty of Engineering, Gifu University Gifu Gifu Japan; ^2^ United Graduate School of Drug Discovery and Medical Information, Sciences Gifu University Gifu Gifu Japan; ^3^ Department of Functional Bioscience Gifu University School of Medicine Gifu Gifu Japan; ^4^ Institute for Glyco‐core Research (iGCORE) Gifu University Gifu Gifu Japan

**Keywords:** anion recognition, anti‐cancer drug, artificial ion channel, calix[4]pyrrole, supramolecular chemistry

## Abstract

The *cis*‐dipyridylcalix[4]pyrrole palladium(II) complex (***cis*‐DPC+Pd^2+^**) forms a cage‐like supramolecular structure that functions as an ion channel, as evidenced by the single ion channel current observations. A lucigenin fluorescence assay revealed that ***cis*‐DPC+Pd^2+^** transports chloride across membranes. The HPTS fluorescence assay revealed that ***cis*‐DPC+Pd^2+^** selectively transports fluoride and chloride. In a cell proliferation assay using THP‐1 cells, the cell survival rate with ***cis*‐DPC+Pd^2+^** was lower than that with ***trans*‐DPC** or ***trans*‐DPC+Pd^2+^**. These results suggest that ***cis*‐DPC+Pd^2+^** causes cell death by forming supramolecular pores, allowing the passage of increased numbers of ions.

## Graphical abstract

The artificial anion channel based on supramolecular calix[4]pyrrole (***cis*‐DPC+Pd^2+^**) was synthesized. This supramolecule exhibited single ion channel currents that repeatedly switched on and off. The lucigenin fluorescence assay revealed that ***cis*‐DPC+Pd^2+^** transports chloride across membranes. The HPTS fluorescence assay showed that ***cis*‐DPC+Pd^2+^** selectively transports fluoride and chloride. A cell proliferation assay using THP‐1 cells suggested that ***cis*‐DPC+Pd^2+^** caused cell death by forming pores with a supramolecular structure, allowing more ions to pass through.

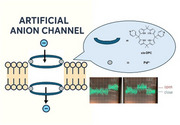

## 1 Introduction

In living organisms, the concentration gradient of inorganic ions (sodium, chloride) across cell membranes is critical for biological phenomena, including membrane potential formation and nerve signal transmission. The cell membrane's phospholipid bilayer maintains this gradient by preventing the passage of charged particles. Conversely, cells possess a mechanism that facilitates ion movement between cell membranes, thereby generating or eliminating concentration gradients as part of their life activities. These mechanisms are mediated by ion transporters (i.e., carriers and channels).

To mimic rapid ion transport across lipid membranes, many artificial cation channels have been developed [1, 2, 3, 4, 5, 6, 7, 8, 9]. However, compared to cation channels, artificial anion channels that allow anions to pass through are more difficult to synthesize because anions have a larger radius than cations, making their synthesis more complex. Various molecules and macrocyclic compounds have been used as scaffolds to form pores (holes) that fit the size of anions [10, 11, 12, 13, 14, 15, 16, 17]. Recent studies show that artificial chloride transporters disrupt intracellular chloride and pH, inhibiting autophagy and inducing apoptosis, suggesting potential as anti‐cancer therapeutics [18, 19, 20]. We are studying supramolecular systems that mimic ion channels by forming large pores with an anion recognition moiety. The present study focuses on transporting chloride in larger quantities and at higher speeds to efficiently induce cell death, making these compounds potential anti‐cancer agents.

Calix[4]pyrrole [21, 22, 23, 24, 25, 26] is a neutral pyrrole‐based anion receptor, but anions cannot pass through its small pore. While some calix[4]pyrrole derivatives have been reported to function as carriers across lipid membranes, they have not been reported to function as ion channels. Previously, we synthesized dipyridylcalix[4]pyrrole bearing two pyridyl groups in the *cis* position and showed that two of these molecules coordinate with Pd^2+^ to form a face‐to‐face cage‐like structure [27]. This supramolecular receptor forms a large anion recognition moiety (i.e., a pore of about 10 Å in diameter); therefore, we expected it to function as an efficient anion channel. Here, we report the results of evaluating the anion channel ability of compound ***cis*‐DPC+Pd^2+^** using electrochemical methods, liposome assays, and a cell‐proliferation‐based cytotoxicity assay.


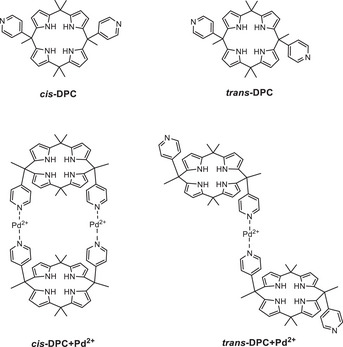


## 2 Results and Discussion

### 2.1 Ion Channel Current Measurement

**FIGURE 1:**
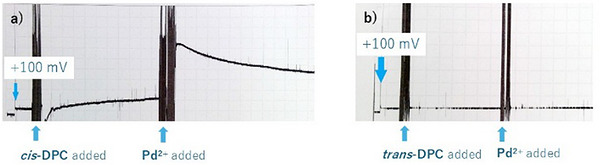
(a) Measurement results after adding ***cis*‐DPC** and then adding the palladium(II) reagent (Vertical axis 0.625 pA/div., horizontal axis 1 min./div., 15 µL of a 5 mM DMSO solution of ***cis*‐DPC** was gently added to both chambers, and then 15 µL of 5 mM DMSO solution of the reagent was added to both chambers and stirred). (b) Measurement results after adding ***trans*‐DPC** and then adding the palladium(II) reagent (Vertical axis 1.25 pA/div., horizontal axis 1 min./div., 15 µL of a 5 mM DMSO solution of ***trans*‐DPC** was gently added to both chambers, and then 15 µL of a 5 mM DMSO solution of the reagent was added to both chambers and stirred).

***Cis‐*DPC**, ***trans‐*DPC**, ***cis*‐DPC+Pd^2+^**, ***trans*‐DPC+Pd^2+^** were evaluated using the planar lipid bilayer membrane method [28, 29] to assess ion transport ability. The bilayer membrane was formed by apposing monolayers at a 100‒200 µm aperture in a Teflon film separating two chambers, each containing 1.5 mL of solution. The monolayer was generated by applying 10 mg/mL diphytanoylphosphatidylcholine in hexane to each aqueous phase and allowing 5 min for solvent evaporation. Prior to membrane formation, the aperture area was coated with 0.5% (v/v) hexadecane in hexane, and the liquid level was raised via tubing. As the solution level rose, the bilayer membrane formed by apposing monolayers at the aperture (see Supporting Information). Unless otherwise specified, both chambers contained 200 mM KCl and 10 mM MOPS/KOH (pH 7.0). Compounds were added to both chambers and transferred to the membrane by stirring. The current across the membrane was measured as voltage via a current‐voltage conversion circuit and displayed on an oscilloscope. When ***cis*‐DPC+Pd^2+^** and ***trans*‐DPC+Pd^2+^** were measured at room temperature, no current was observed for ***trans*‐DPC+Pd^2+^**. However, ***cis*‐DPC** increased current upon introduction of the Pd(II) reagent ([Pd(II)(OTf)_2_(PEt_3_)_2_]) (Figure 1).

**FIGURE 2:**
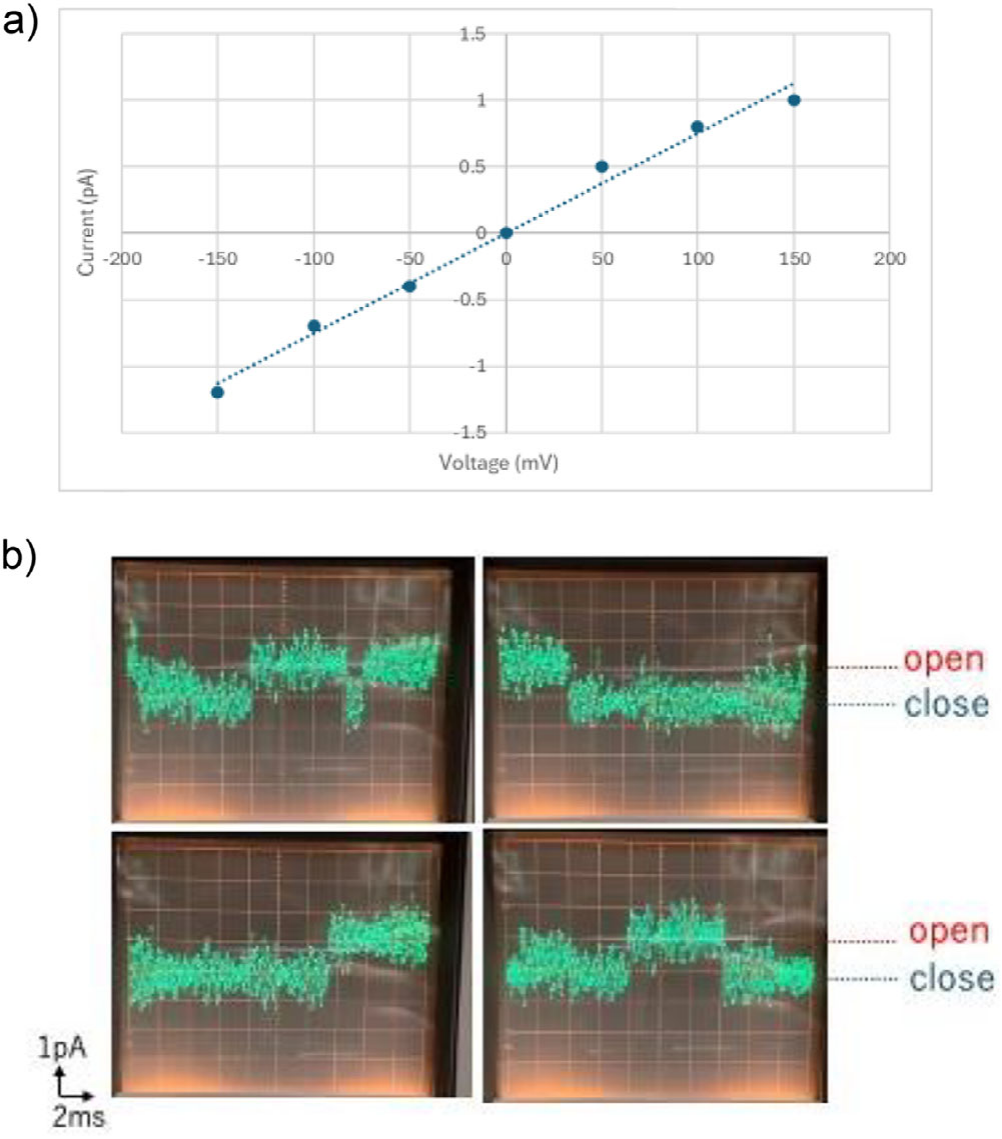
(a) Current values when ***cis*‐DPC+Pd^2+^** was added, and the voltage was changed (***cis*‐DPC+Pd^2+^** was dissolved in hexane together with lipid, and 20 µL was added to each of the two water phases. After the membrane had formed, the voltage was changed to ±150, ±100, and ±50 mV, and the magnitude of the current flow was measured. The ***cis*‐DPC** concentration was 0.21 mM, the lipid concentration was 31 mM, and the lipid‐to‐***cis*‐DPC** molar ratio was 148:1). (b) Observed single ion channel current.

***Cis*‐DPC+Pd^2+^** caused larger currents than ***cis*‐DPC** in current measurement using the lipid bilayer membrane method, suggesting pore formation with a supramolecular structure and channel function. However, a single ion channel current with repeated on and off states was not confirmed. Therefore, the carrier and channel properties were assessed via another method. In the conventional method, we added compounds dissolved in DMSO to the aqueous phase after forming the lipid bilayer membrane, waited for spontaneous migration to the membrane, and measured the current. However, this approach had limitations: (i) slow compound transfer to the membrane, (ii) compound precipitation in the aqueous phase, and (iii) lipid membrane breakage when stirred. Furthermore, the number of molecules transferred to the membrane was unclear. We attempted to solve these problems by pre‐mixing lipids and compounds prior to forming the lipid bilayer membrane. ***cis*‐DPC+Pd^2+^** was dissolved in hexane with lipids, 20 µL of this solution was added to each aqueous phase, and the monolayers were apposed to form a bilayer membrane. The applied voltage varied (±150 mV, ±100 mV, and ±50 mV), and the current magnitude was recorded. Measurements of ***cis*‐DPC+Pd^2+^** were performed at 0.21 mM. As seen in Figure 2a, ***cis*‐ DPC+Pd^2+^** caused larger currents than lipid alone. Thus, ***cis*‐DPC +Pd^2+^** exhibited ion transport function when lipids and compounds were pre‐mixed to form the lipid bilayer membrane. When applying +150 mV, discrete stepwise conductance fluctuations were observed (Figure 2b). These single ion channel currents repeated on and off (Figure 2b) suggested that ***cis*‐DPC+Pd^2+^** formed pores (holes) within the lipid bilayer membrane and functioned as channels allowing ion passage^1^. The state of the on/off behavior of the single ion channel current could no longer be confirmed as time passed (approximately 1 h). The Pd^2+^ part of the supramolecular structure carries an electric charge and is therefore drawn into the aqueous phase, leading to structural distortion.

### 2.2 Lucigenin Fluorescence Assay

**FIGURE 3:**
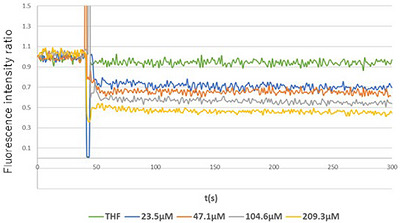
Lucigenin fluorescence assay (λex = 370 nm, λem = 500 nm).

A single ion channel current was observed, indicating that an ion channel had formed, but since KCl was dissolved in both aqueous chambers, it was unclear whether either K^+^ or Cl^−^ was passing through the lipid bilayer membrane. Therefore, we performed a lucigenin assay using the liposome method [30]. As Figure 3 shows, the addition of THF to the lucigenin‐containing liposomes did not change the fluorescence intensity, suggesting that THF did not affect the liposomes. Fluorescence quenching was confirmed by adding ***cis*‐DPC+Pd^2+^**. This was attributed to chloride entering the liposome and reacting with lucigenin. The rapid quenching suggested that the compound formed transient pores in the membrane. Fluorescence quenching increased with increasing concentration. This was attributed to an increase in the amount of compound incorporated into the membrane.

### 2.3 HPTS Assay

**FIGURE 4:**
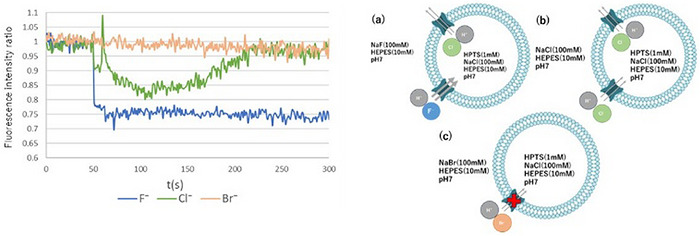
HPTS fluorescence assay (***cis*‐DPC+Pd^2+^** in THF solution: 250 µM, λex = 405 nm, λem = 510 nm).

Next, the HPTS assay [31] was conducted to investigate anion selectivity. This fluorescence‐based assay revealed a decrease in the fluorescence intensity ratio upon the addition of ***cis*‐DPC+Pd^2+^** to liposomes in the presence of various external anions (Figure 4). When fluoride (F^−^) was present outside the liposome, the fluorescence quenching was most significant. This behavior indicated that fluoride was transported more efficiently than chloride (Cl^−^) (Figure 4a). A decrease in intensity was also observed when chloride was present outside the liposome, followed by recovery and a return to the same intensity as before ***cis*‐DPC+Pd^2+^** was added. This occurred because the H^+^ concentration gradient was gradually dissipated due to the permeability of the liposome^2^ (Figure 4b). No change in fluorescence intensity was observed when bromide (Br^−^) was present outside the liposome. This was attributed to the low binding affinity of ***cis*‐DPC+Pd^2+^** for bromide (Figure 4c). These results suggested that ***cis*‐DPC+Pd^2+^** may form supramolecular pores within the membrane in conjunction with the Pd(II) reagent, functioning as anion‐selective channels^3^ that promote increased ion flux.

### 2.4 Cell Proliferation Assay

**FIGURE 5:**
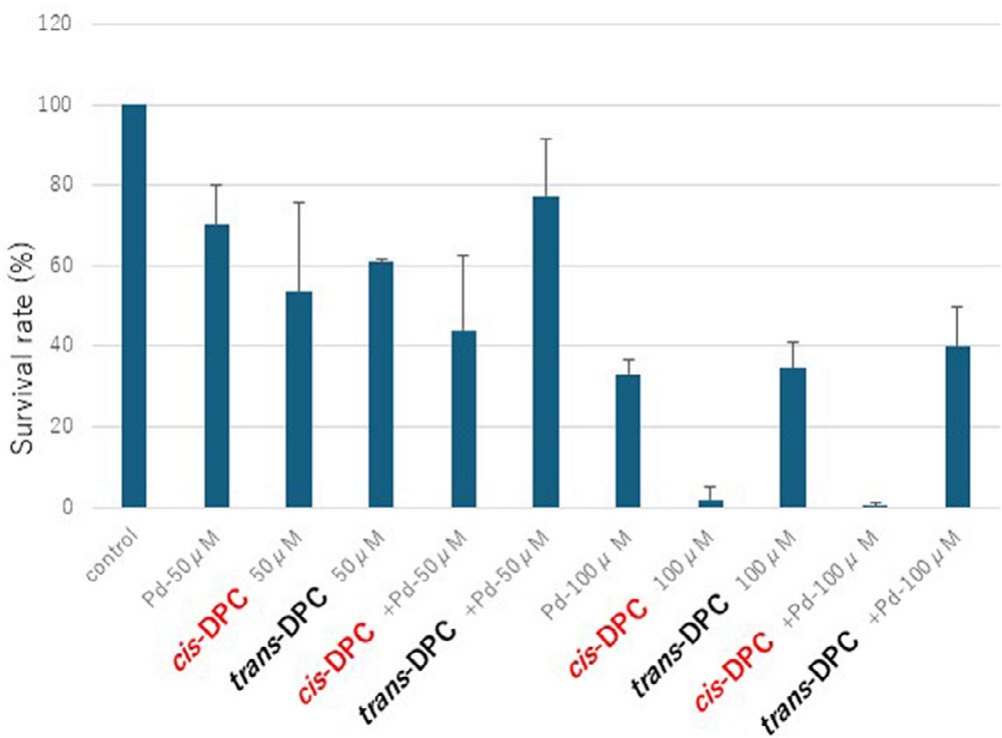
Average survival rate (%) and its standard deviation when each treatment was administered to cells. This experiment used THP‐1 cells, a human monocyte cell line. Cells were cultured at 37°C in the presence of 5% CO_2_ in RPMI 1640 medium supplemented with 10% FBS (fetal bovine serum), 100 µg/mL streptomycin sulfate, 20 U/mL penicillin G potassium, and 10 mM HEPES.

Based on these results, we investigated whether there was a difference in the ability to induce THP‐1 cancer cell death between ***cis*‐DPC+Pd^2+^**, which passed an ionic current, and ***trans***‐DPC+Pd^2+^, which did not. The results shown in Figure 5 reveal that the cell survival rate was lower when ***cis*‐DPC** was administered in the cell proliferation assay [32] compared to when ***trans*‐DPC** was administered. Furthermore, when ***cis*‐DPC** was administered to cells together with the palladium(II) reagent, the survival rate was lower than in the absence of that reagent. Thus, ***cis*‐DPC+Pd^2+^** produced the most significant cell death^4^.

**FIGURE 6:**
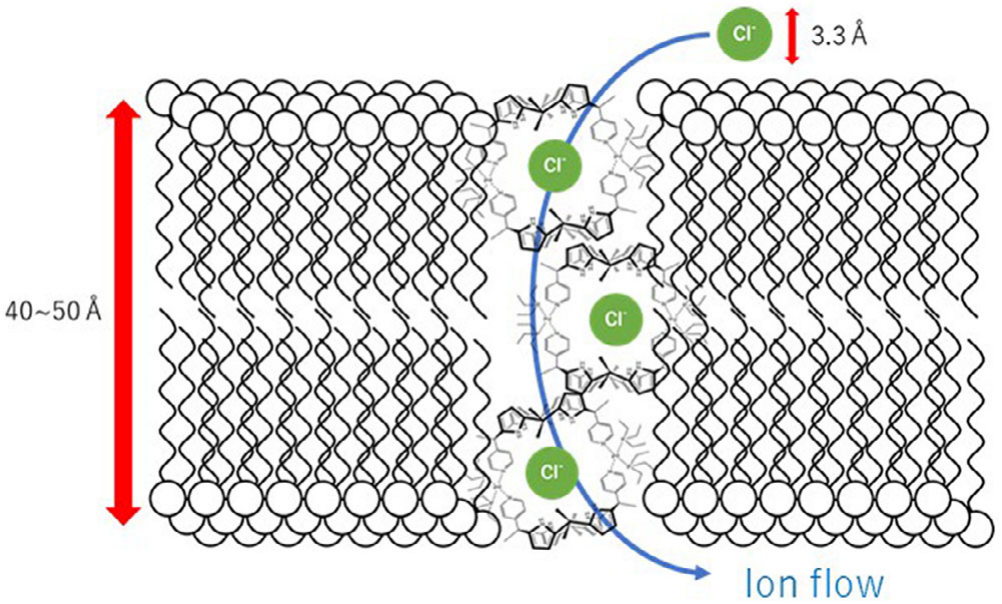
Schematic representation of chloride ions passing through lipid membranes.

The size of the supramolecular calix[4]pyrrole ***cis*‐DPC+Pd^2+^** is about 10 Å, while the lipid bilayer thickness is about 40‒50 Å. Therefore, it is considered that two or more molecules of ***cis*‐DPC+Pd^2+^** entered the upper and lower monolayers of the bilayer membrane and then overlapped in these layers by lateral diffusion, creating pores and allowing ions to pass through (Figure 6)^5^.

## 3 Conclusion

In conclusion, in the planar lipid bilayer membrane method, a significant current flow between chambers separated by a lipid bilayer membrane was detected when ***cis*‐DPC** and the palladium(II) reagent were mixed in the chamber. This suggested that ***cis*‐DPC** and the palladium(II) reagent together form a supramolecular structure (***cis*‐DPC+Pd^2+^**) that allows ions to pass through the lipid bilayer membrane. In a cell proliferation assay, THP‐1 cells treated with mixtures of ***cis*‐DPC** or ***trans*‐DPC** and their respective palladium(II) reagents showed that the cell survival rate with ***cis*‐DPC+Pd^2+^** was lower than that with ***trans*‐DPC+Pd^2+^**, in which no ionic current was detected. These results suggested that the combination of ***cis*‐DPC** and the palladium(II) reagent causes cell death by forming pores through the supramolecular structure ***cis*‐DPC+Pd^2+^**, allowing more ions to pass through. Furthermore, ***cis*‐DPC+Pd^2+^** was found to function as an ion channel because a single ion channel current was observed. The lucigenin fluorescence assay revealed that ***cis*‐DPC+Pd^2+^** transports chloride across membranes. ***cis*‐DPC+Pd^2+^** was also found to selectively transport fluoride and chloride, with fluoride transport being the most dominant in the HPTS fluorescence assay.

## Conflicts of Interest

The authors declare no conflicts of interest.

## Supporting information



Supporting information is available for synthesis, lipid bilayer membrane method, lucigenin fluorescence assay, HPTS assay, and cell growth activity.
**Supporting File 1**: asia70905‐sup‐0001‐SuppMat.docx

## Data Availability

The data that supports the findings of this study are available in the supplementary material of this article

## ENDNOTES

1 It is possible to determine the conductivity from the area of the planar lipid bilayer. Since 1 pA flowed at 150 mV, the conductance (G) is approximately 6.67 pS. The conductivity (σ) is approximately 1.06 pS/m for a hole with a radius of 100 µm, assuming a lipid bilayer thickness of approximately 50 Å. This value is thought to be close to that observed in natural ion channels.

2 Since this phenomenon is not observed for F^−^ and Br^−^, it is suggested that dipyridylcalix[4]pyrrole, upon recognizing Cl^−^, attempts to maintain equilibrium as a good proton carrier within the liposome membrane.

3 Generally, the affinity of calix[4]pyrrole for anions is F ^−^> Cl ^−^> Br^−^, so this result suggests that the calix[4]pyrrole moiety is functioning as an ion‐selective filter.

4
***Cis*‐DPC** also seems to function as a carrier since it reduces the cell proliferation rate and increases the current value as the concentration of ***cis*‐DPC** increases in the lipid bilayer membrane method (see Figure S2).

^5^
It is difficult to know precisely what kind of multi‐assembly channel is formed, but in the method of adding Pd^2+^ later, as shown in Fig. 1, a large current value was obtained when Pd^2+^ was added to both chambers, which suggests that pores are probably formed near the water surface on both sides of the lipid bilayer, as shown in Fig. 6. Furthermore, the method in Fig. 1 suggests that multiple channels are open simultaneously, while the method in Fig. 2 suggests that a single channel is open.
