# Supplementary material for: An Artificial Anion Channel Based on Supramolecular Calix[4]Pyrrole and Its Application as an Anti‐Cancer Agent
Source: Chem Asian J. 2026 Jul 21;21(14):e70905. doi: 10.1002/asia.70905 (PMC13387506; doi:10.1002/asia.70905)
Supplement: Supplementary file 1 — Supporting information is available for synthesis, lipid bilayer membrane method, lucigenin fluorescence assay, HPTS assay, and cell growth activity. Supporting File 1: asia70905‐sup‐0001‐SuppMat.docx [file ASIA-21-e70905-s001.docx]

Supporting Information
©Wiley-VCH 2016
69451 Weinheim, Germany

An artificial anion channel based on supramolecular calix[4]pyrrole and its application as an anti-cancer agent

Hidekazu Miyaji,* Yuki Shiozawa, Takahiro Matsubara, Tomoyoshi Terada, and Yoshinori Muto

Abstract: The *cis*-dipyridylcalix[4]pyrrole palladium(II) complex (*cis*-DPC+Pd^2+^) forms a cage-like supramolecular structure that functions as an ion channel, as evidenced by single ion channel current observations. A lucigenin fluorescence assay revealed that *cis*-DPC+Pd^2+^ transports chloride across membranes. The HPTS fluorescence assay revealed that *cis*-DPC+Pd^2+^ selectively transports fluoride and chloride. In a cell proliferation assay using THP-1 cells, the cell survival rate with *cis*-DPC+Pd^2+^ was lower than that with *trans*-DPC or *trans*-DPC+Pd^2+^. These results suggest that *cis*-DPC+Pd^2+^ causes cell death by forming supramolecular pores, allowing the passage of increased numbers of ions.

Experimental Procedures

**General information**

All reagents and solvents were obtained from commercial sources and used without further purification unless otherwise noted. ^1^H NMR spectra (ECA-500/ECS-400/JEOL), fluorescence spectra (RF-5300PC/SPECTROFLUOROPHOTOMETER/SHIMADZU), FAB-MS (JMS-MStation 700), and ESI-MS (JMS-T100LC AccuTOF™/JEOL) were recorded on the indicated instruments.

**Synthetic details**

5-methyl-5-(4-pyridyl)dipyrromethane^1^

4-Acetylpyridine (2.6 mL, 24 mmol) and pyrrole (4.5 mL, 75 mmol) were suspended in water (160 mL) and the mixture was stirred with hydrochloric acid for 1 h at room temperature. After neutralization with sodium bicarbonate, 5-methyl-5-(4-pyridyl)dipyrromethane was precipitated and dried *in vacuo*. The residue was further purified by silica column chromatography (1.1 g, 19%). ^1^H NMR (500 MHz, CDCl_3_) δ 2.03 (s, 3H, CH_3_), 5.95 (m, 2H, pyrrole-CH), 6.18 (m, 2H, pyrrole-CH), 6.70 (m, 2H, pyrrole-CH), 7.02 (d, 2H, pyridine-CH), 7.82 (s, 2H, pyrrole-NH), 8.50 (d, 2H, pyridine-CH). FAB-MS m/z = 238.2 (M+H^+^).

5,15-dipyridyl-5',10,10',15',20,20'-hexamethylcalix[4]pyrrole^1^

5-Methyl-5-(4-pyridyl)dipyrromethane (300 mg, 1.3 mmol) was dissolved in acetone (500 mL). BF_3_•OEt_2_(0.5 mL, 4.1 mmol) was added to the solution and stirred for 24 h. After neutralization with triethylamine, the solution was concentrated *in vacuo*, and the mixture of CHCl_3_/water was poured. The organic layer was separated, washed with water, and dried *in vacuo*. The residue was purified by silica column chromatography (eluent: chloroform : ethyl acetate = 1 : 2) (31 mg, 9% (Rf 0.23: *cis*), 84 mg, 24% (Rf 0.33: *trans*)). ^1^H NMR (400 MHz, CD_3_CN) δ 1.47 (s, 6H, CH_3_), 1.62 (s, 12H, CH_3_), 5.62 (d, 4H, pyrrole-CH), 5.84 (d, 4H, pyrrole-CH), 6.82 (d, 4H, pyridine-CH), 7.82 (s, 4H, NH), 8.38 (d, 4H, pyridine-CH). ESI-MS m/z = 555.1120 (M+H^+^).

**Palladium reagent: [Pd(II)(OTf)₂(PEt₃)₂]^2^**

*Trans*-dichloro bistriethylphosphine palladium(II) (82.1 mg, 0.198 mmol) and silver trifluoromethanesulfonate (111.5 mg,0.434 mmol) were added to 10 mL of dichloromethane and reacted overnight under nitrogen. The reaction solution was filtered through celite and concentrated by evaporation. Hexane was added to reduce the solubility, yielding needle-like crystals. The crystals were collected by suction filtration, yielding 14.1 mg, 10.1% yield. ¹HNMR (400 MHz, CD₂Cl₂), δ 2.06-1.98 (m, 12H, CH₂), 1.38-1.29 (m, 18H, CH₃).

*cis*-dipyridylcalix[4]pyrrole palladium(II) complex (*cis*-DPC+Pd^2+^)

*Cis*-5,15-dipyridyl-5',10,10',15',20,20'-hexamethylcalix[4]pyrrole (4.5 mg, 0.008 mmol) and the palladium reagent (35.1 mg, 0.008 mmol) were dissolved in dichloromethane, reacted for 1 h, and then concentrated using an evaporator. ¹HNMR(400MHz, CD₃CN) **δ**1.18(m, 36H, ethyl-CH3), 1.49(s, 12H, CH3), 1.63(s, 24H, CH3), 1.98(m, 24H, P-CH2), 5.34(d, 8H, pyrrole-CH), 5.93(d, 8H, pyrrole-CH), 7.02(d, 8H, pyridine-CH), 7.68(s, 8H, NH), 8.55(d, 4H, pyridine-CH). ESI-MS m/z=2389.22(M+H⁺).

Results and Discussion

Lipid bilayer membrane method

Compounds were dissolved in hexane with lipids, and 20 µL of each solution was added to two water baths. After bonding the membranes together, the current flow was measured at voltages of ±150 mV, ±100 mV, ±80 mV, ±60 mV, ±40 mV, and ±20 mV. Measurements were performed at compound concentrations of 0.18 mM, 0.36 mM, and 0.90 mM (the lipid concentration was 31 mM, corresponding to lipid-to-compound molar ratios of 172:1, 86:1, and 34:1).


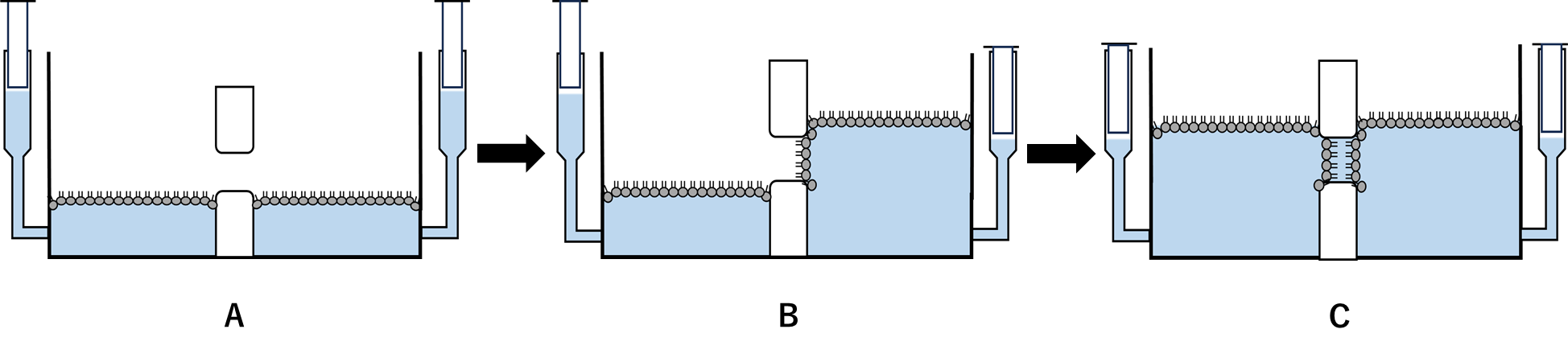


**Figure S1** Preparation of lipid bilayer membrane: (A) Place a buffer solution into both chambers, add hexane containing dissolved lipids, and form a monolayer on the water surface. (B) Slowly add buffer solution into the chamber via a syringe to raise the water level. (C) When the water level rises above the small hole, a bilayer is formed.

**Movie file of single ion channel current measurement（Compound: *cis*-DPC+Pd^2+^, Voltage: +150 mV）**

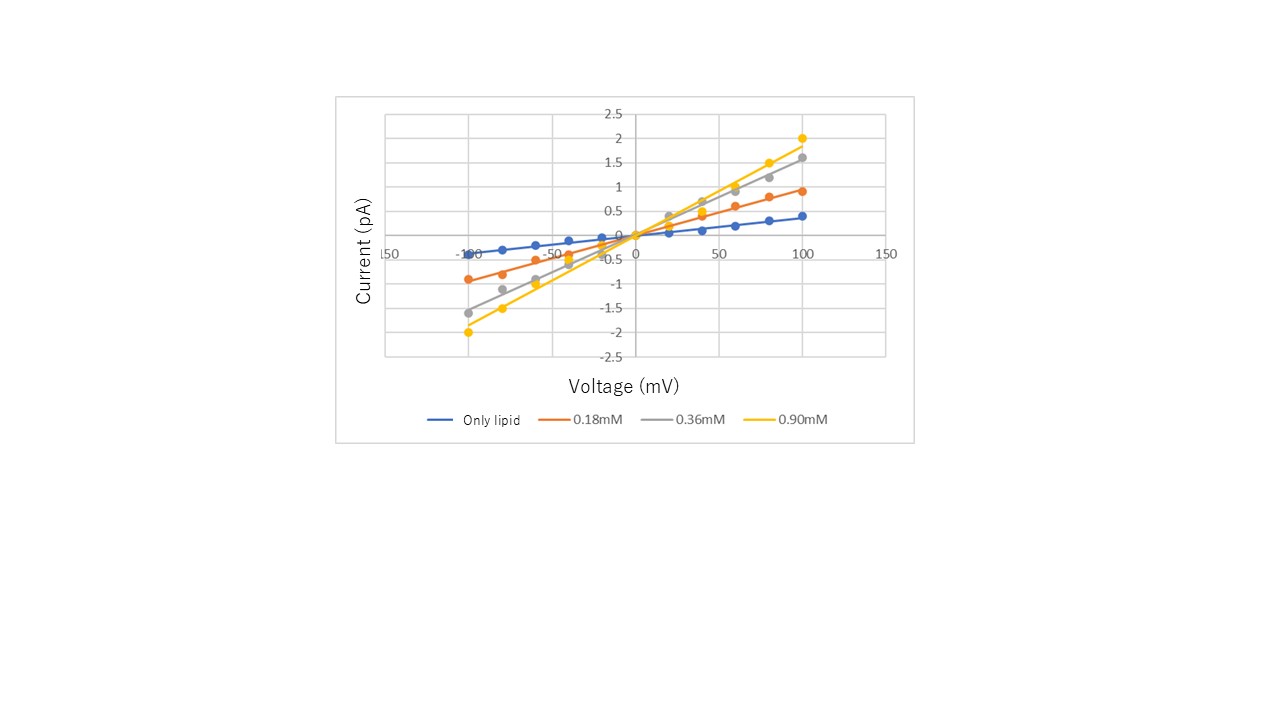


**Figure S2** Current values when ***cis*-DPC** was added and the voltage was changed (***cis*-DPC** was dissolved in hexane together with lipid, and 20 µL was added to each of the two water phases. After membrane formation, the voltage was changed to ±100mV, ±80 mV, ±60 mV, ±40 mV, and ±20 mV, and the magnitude of the current flowing was measured. ***Cis*-DPC** concentrations were 0.18 mM, 0.36 mM, and 0.90 mM (the lipid concentration was 31 mM, and the lipid-to-***cis*-DPC** molar ratios were 172:1, 86:1, and 34:1, respectively).

**Lucigenin fluorescence assay**

In a 100 mL recovery flask, lecithin (25 mg) was dissolved in EtOH/CHCl₃ (2 mL, v/v 1:1). This solution was rotary evaporated to obtain a thin film, which was then vacuum dried for 1 day to remove residual solvent. To this lipid film, 2.0 mL of buffer (10 mM MOPS + 100 mM KNO₃ + 1 mM lucigenin, pH = 7.0) was added, and the thin film was peeled off using a vortex mixer. The resulting solution was then sonicated for 20 m (Figs. 3‒4). The resulting liposome solution was subjected to gel filtration using a desalting mini-column, Ampure^TA^ SA (eluent: 10 mM MOPS + 100 mM KNO₃, pH = 7.0). A 50 μL solution of lucigenin-containing liposome was suspended in 1950 μL of buffer (10 mM MOPS + 100 mM KCl, pH = 7.0) and placed in a quartz cuvette. THF or a THF solution of compound 2 was added at t = 40 s, and the lucigenin fluorescence intensity was measured over time.

**HPTS assay**

In a 100 mL eggplant flask, lecithin (25 mg) was dissolved in EtOH/CHCl₃ (2 mL, v/v 1:1). The solution was rotary evaporated to obtain a thin film, which was then vacuum dried for 3 days to remove residual solvent. Two milliliters of buffer (10 mM HEPES + 100 mM NaCl + 1 mM HPTS, pH = 7.0) was added to the lipid film, and the thin film was peeled off using a vortex mixer. The resulting solution was then sonicated for 20 min. The resulting liposome solution was subjected to gel filtration using an Ampure^TA^ SA desalting mini-column (eluent: 10 mM HPTS + 100 mM NaCl, pH = 7.0). A 50 μL solution of HPTS-loaded lecithin liposomes was suspended in 1950 μL buffer (10 mM HEPES + 100 mM NaX, X = Cl, Br, F, NO_3_, pH = 7.0) and placed in a quartz cuvette. THF and a THF solution of the compound were added at t = 50 s, and the fluorescence intensity was measured over time.

**Cell lines, cell culture, and cell growth activity**

Cells of the human monocytic cell line THP-1 were maintained in RPMI 1640 medium with L-glutamine, supplemented with 10% FBS, 100 μg/mL streptomycin sulfate, 20 U/mL penicillin G potassium, and 10 mM HEPES, at 37 ℃ and 5% CO_2_ in a humidified incubator. For the cell growth assay, THP-1 cells were seeded in triplicate in 96-well plates at 1 × 10^4^ per well in 100 μL of RPMI 1640 medium with L-glutamine, supplemented with 10% FBS and Pd, *cis*-DPC, *trans*-DPC, *cis*-DPC + Pd or *trans*-DPC + Pd (final concentration of 0, 50, or 100μM). Cells were incubated for 24 h at 37 ℃ and 5% CO_2_. Ten microliters of the cell proliferation reagent WST-1 was added to the cells in 96-well plates, followed by incubation for 4 h at 37 ℃ and 5% CO_2_. The plate was read on a Model 680 Microplate Reader (Bio-Rad Laboratories, Hercules, CA, USA) at 450 nm with a reference wavelength of 655 nm.

**―――――――――――――――――――――――――**

**Reference**

1. N. Kiriyama, M. Ebihara, T. Udagawa, H. Miyaji, *RSC Adv.* **2016**, 6, 19794-19796.

2. J. López-Serrano, S.B. Duckett, A. Lledós, *J. Am. Chem. Soc.* **2006**,128(30),9596-9597.

# Author Contributions

H.M. conceived the project. H.M. prepared the manuscript and the supporting information. Y.M., Y.S. and T. M. evaluated electrochemical properties. Y.S. studied Lucigenin assay and HPTS assay. T.T. studied cell proliferation assay using THP1 cells.
